# Development of an In-Situ Multifrequency Electromagnetic Sensor for Real-Time Microstructure Monitoring in a Continuous Annealing Furnace

**DOI:** 10.3390/s25165158

**Published:** 2025-08-19

**Authors:** John W. Wilson, Mohsen A. Jolfaei, Lei Zhou, Carl Slater, Claire Davis, Anthony J. Peyton

**Affiliations:** 1 Department of Electrical and Electronic Engineering, University of Manchester, Manchester M13 9PL, UK; a.peyton@manchester.ac.uk; 2 Warwick Manufacturing Group, University of Warwick, Coventry CV4 7AL, UK; mohsen.jolfaei@warwick.ac.uk (M.A.J.); lei.zhou@warwick.ac.uk (L.Z.); c.d.slater@warwick.ac.uk (C.S.); claire.davis@warwick.ac.uk (C.D.)

**Keywords:** electromagnetic sensor, non-destructive testing, steel, magnetic properties, annealing, high temperature

## Abstract

The continuous annealing process is widely used in the production of advanced high-strength steels. However, to tightly regulate the mechanical properties of the steel, precise control of processing parameters is needed. Although some techniques are available to monitor the mechanical properties of the steel on entry and exit to the furnace, monitoring the evolving microstructure of the steel through installation of sensors in the annealing line is extremely challenging due to the high temperature, high speed of the steel strip and limited space in the furnace. This study presents the development and validation of a multifrequency electromagnetic sensor system for real-time monitoring of microstructural transformations in steel during thermal cycling, intended for deployment in a continuous annealing line. Experiments were conducted on austenitic stainless steel to study the signal response to an increase in resistivity without a change in magnetic permeability. Pure nickel was tested to investigate the response to a change in magnetic permeability and the ferromagnetic-to-paramagnetic transition at its Curie temperature. A ferritic stainless steel was also tested to assess the performance of the system for high-temperature ferromagnetic materials and a higher-temperature ferromagnetic-to-paramagnetic transition. The tests indicate a strong response to material resistivity and permeability changes, with complementary information from different frequencies. Test results are supplemented by a finite element modelling study into the effect of a change in frequency and permeability on sensor response, with a discussion on the implications of experimental and modelling results for future applications. The results show that the developed system has the potential to characterise thermally induced changes in steels, establishing proof of concept for non-destructive, high-temperature electromagnetic sensing in steel processing and setting the foundation for further industrial deployment in phase and recrystallisation monitoring.

## 1. Introduction

Continuous annealing furnaces provide a controlled thermal environment for adjusting steel microstructure post cold-rolling. The continuous annealing process (CAP) subjects the cold-rolled material to a controlled heating and cooling sequence to improve its properties, such as ductility and strength, by passing the material through a series of gas or induction furnaces and cooling sections. Unlike other industrial annealing processes, CAP is a non-equilibrium, rapid process that lasts for approximately 60–120 s and is therefore strongly influenced by the kinetics of the metallurgical process of interest (for example, austenitisation, recrystallisation, phase transformation) [1]. CAP enables precise control of heating and cooling rates in a high-throughput manner, making it ideal for the production of advanced steels such as dual-phase and interstitial free grades [2,3].

Given the need for precise process control to produce high-value steels in CAP, in situ characterisation methods that can reliably track evolving microstructures to ensure mechanical property targets are much sought after. It is, however, a challenging environment with high temperatures, limited space, fast-moving steel strip and a large lift-off (distance between sensor and strip) to contend with. Current techniques for the determination of continuous annealing process parameters involve a combination of mathematical, physical and microstructural models to inform process parameters along with some online measurements from strip speed and tension and a limited number of pyrometers for temperature measurement, [4]. Additional information can be gained from employing a system such as IMPOC, which uses remanent magnetisation to obtain the material’s tensile strength and yield strength during production [5]. However, IMPOC is typically only employed on entry or exit from the line, not during the annealing process, as it cannot be used within a high-temperature environment. One system is available that permits the steel structure to be determined within the annealing process. It uses X-ray diffraction to define the crystalline phase fraction, and hence the austenite content of the material [6]. However, it is not widely deployed due to line-of-sight constraints, high-temperature interference, and the need for radiation shielding.

Electromagnetic (EM) sensors have demonstrated significant potential in characterising the microstructure of steel, both in offline and online applications [7]. EM techniques provide valuable insights into heat-induced microstructural changes in metals, offering a complementary approach by leveraging changes in magnetic properties and electrical resistivity to deliver real-time information on microstructural evolution. In the lab, EM sensors have been used to study phenomena such as recovery and recrystallisation [8], mechanical properties [9] and magnetic anisotropy [10]. In [8] changes in the microstructural state of an interstitial free steel caused by recovery and recrystallisation during heat treatment are detected using an EM sensor within the furnace. The measurement coils are formed around a ceramic cylinder, with the sample placed in the cylinder for measurement. This configuration eliminates problems with lift-off as the entire sample is within the measurement coil, so it can give very accurate results. However, it cannot be used on open plates or strip samples, so it is not suitable for industrial installations.

The use of multifrequency excitation in electromagnetic non-destructive testing is well established, with publications dating back to the 1960s [11,12]. Utilising the skin depth effect [13], the material under inspection can be probed at several depths simultaneously [14], offering some key advantages over single-frequency applications [15]. Defects in metals can be analysed in more detail, allowing the characterisation of flaws with more complex shapes [16] and improving defect characterisation capabilities in the presence of variations in conductivity, permeability, geometry and probe lift-off by subtracting the characteristic signals of these variations [17]. Multifrequency testing is also advantageous where the material property of interest varies with depth, for example, in laser hardened steel [18], for the characterisation of residual stresses [19] and for the identification of bimetallic coins [20].

Some EM measurement systems have been developed for industrial applications: IMPOC, HACOM, and 3MA are already widely used in online settings like strip steel production with signals correlated with mechanical properties [21]. For real-time microstructure monitoring, the EMspec™ system has been developed to track phase transformations during hot strip steel processing, where the sensor is located close to the hot strip but is water-cooled and therefore does not need to be designed from high-temperature resistant materials [22]. However, for CAP installation, the sensor assembly needs to be located in the hot zone and exposed to temperatures in the range of 650–850 °C. This is not possible for the current commercial systems, as they are complex constructions employing components such as ferrite cores, which would not survive continued exposure to high temperatures. For CAP installation a simplified approach is needed, with only the sensing coils and minimal supporting structure located in the hot zone and all other system components located externally to the furnace.

This study details the development of a multifrequency EM measurement system for deployment in a continuous annealing furnace. The primary aim of the work is to demonstrate the capability and robustness of the sensor system in tracking temperature-dependent changes in permeability and conductivity under high-temperature conditions. Although transformation phenomena are not directly studied, different materials are tested to investigate different aspects of the sensor response, with the scope of the paper centred on sensor design and its functional viability within CAP environments.

## 2. Materials and Methods

### 2.1. Experimental Test Rig

**Figure 1:**
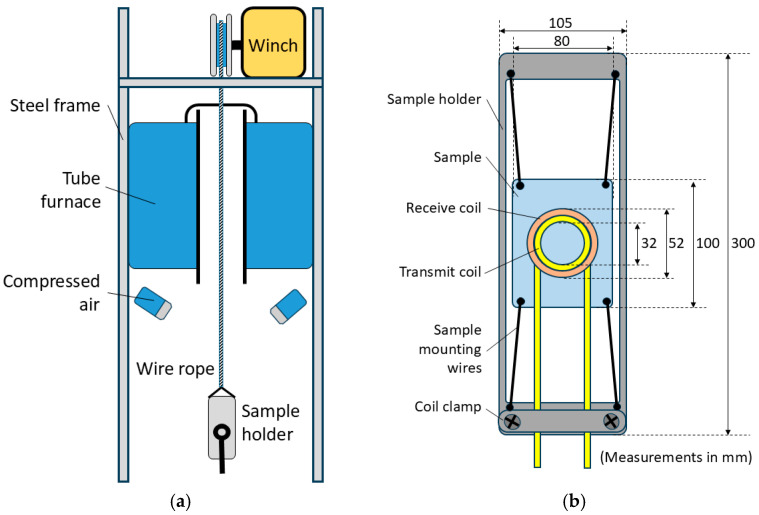
Diagram of the experimental rig (**a**) and diagram of the sample holder and coil (**b**).

Figure 1a shows a diagram of the annealing simulator test rig built by the University of Warwick. A Carbolite Gero (Hope Valley, UK) HTRV vertical tube furnace is mounted in a type 304 stainless steel frame with a winch positioned above it to allow the sample holder (Figure 1b) to be moved in and out of the furnace using a wire rope. The tube furnace has a maximum temperature of 1800 °C. The user has the option of slowly cooling the sample in the furnace, lowering the sample out of the furnace for faster cooling in air or forced cooling using the compressed air jets. The sample holder was laser cut from a sheet of 4 mm austenitic stainless steel and allows the measurement coils to be clamped in place, close to the sample surface, with the sample mounted in the centre of the sample holder, with short lengths of thermocouple wire holding it in place.

**Figure 2:**
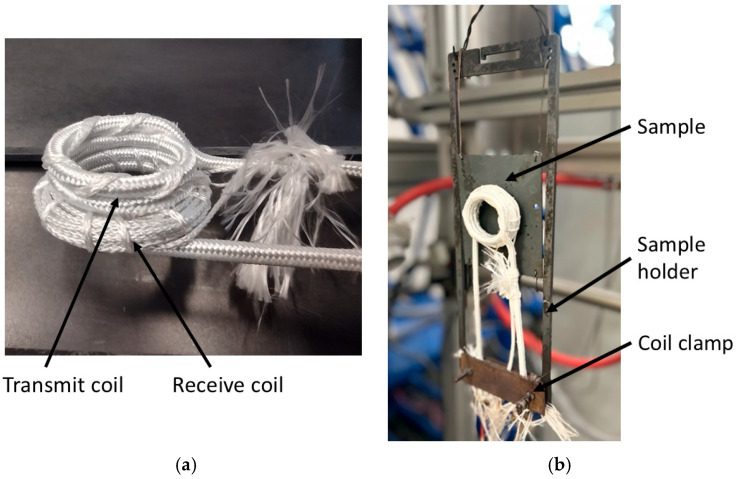
Photograph of the sensor assembly (**a**) and photograph of the sample holder and coil assembly (**b**).

Figure 2a shows the coil assembly. A commercially produced induction heating coil [23] is used for excitation. The coil is made from 3.5 turns of 4 mm solid copper rod and was re-sleeved using alumina silica fibre sleeving for increased temperature resistance. It also provides a readily available, rigid structure to act as a former for the receive coil. A 12-turn receive coil, made from 0.8 mm chromel wire taken from a K-type thermocouple extension cable, was wrapped around the outside of the transmit coil and secured using thermocouple sleeving. A photograph of the sample holder with sample and coil assembly is shown in Figure 2b.

**Figure 3:**
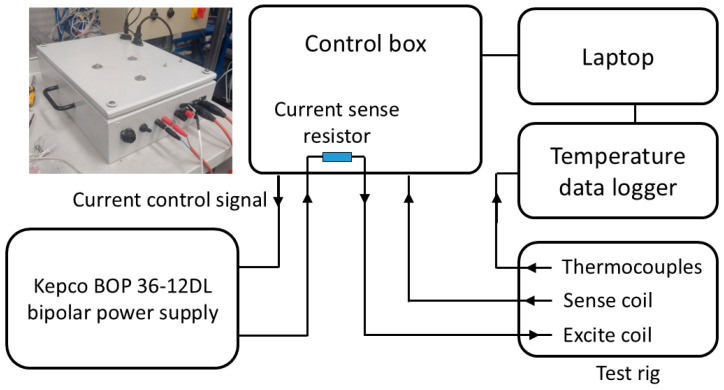
Block diagram of the EM measurement system.

A block diagram of the EM measurement system developed at the University of Manchester is shown in Figure 3. The measurement system consists of a custom-built control box, which provides a multifrequency excitation voltage to a Kepco (Flushing, NY, USA) 400-Watt Bipolar Operational Power supply (BOP). The output current of the BOP is proportional to the input voltage; therefore, the excitation current remains the same throughout the test, irrespective of thermally induced changes in transmit coil resistance. The receive coil is interfaced with the control box, which amplifies the coil voltage and records the signal using a National Instruments (Austin, TX, USA) data acquisition device.

K-type thermocouples were used to record the temperature of the coil and the sample at various positions throughout the tests. Data was acquired simultaneously from the EM measurement system and the K-type thermocouples using a Pico Technology (St Neots, UK) temperature data logger. Temperature and EM signals were recorded throughout the experiments, with the recorded temperature and time interpolated and synchronised to align the temperature with the corresponding EM data.

The EM measurement system can apply a range of excitation frequencies simultaneously. LabVIEW (Version 21.0) software is used to generate the multifrequency excitation signal, while extracting amplitude and phase values from the receive coil signal for each frequency using a windowed fast Fourier transform. Amplitude and phase values are plotted in real time and recorded. Data is sampled at a rate of 1 MS/s with one frame of data acquired every 100 ms.

### 2.2. Excitation Parameter Selection

**Figure 4:**
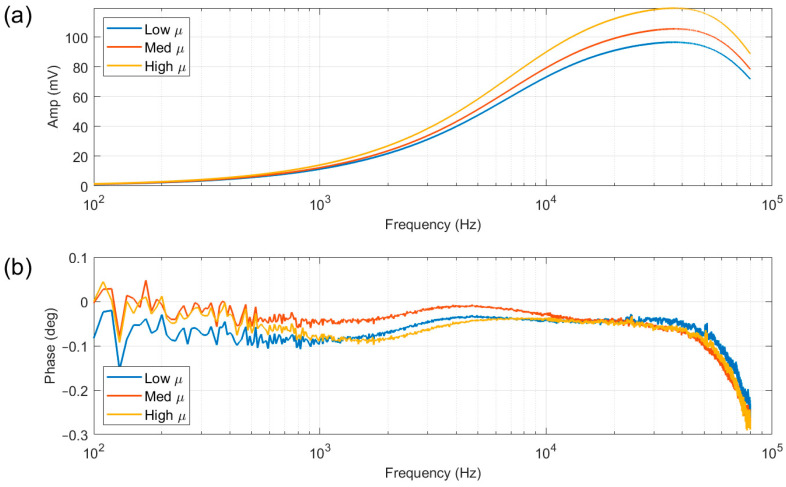
Amplitude (**a**) and phase (**b**) readings resulting from a frequency sweep from 100 Hz to 80 kHz with ferrite plates with low, medium (med), and high permeability values.

A test was set up to determine the optimal excitation parameters for the tests using plates made from ferrite powder in a ceramic or polymer medium. This type of material was selected as it has a clearly defined magnetic permeability, along with a very high resistivity (>200 Ω/cm), which allows the assessment of the frequency response of the system to a variation in magnetic permeability without a significant contribution from electrical resistivity. The ferrite plates have known initial relative permeabilities of 230 (Low μ) [24], 650 (Med μ) [25] and 3000 (High μ) [26] and were tested with a lift-off of 3 mm. A frequency sweep from 100 Hz to 80 kHz was applied to the three samples, and an air measurement (response with no sample present) was subtracted. The amplitude response (Figure 4a) shows clear discrimination between the three samples, with the higher permeability sample producing a higher amplitude response throughout the frequency spectrum. Conversely, the phase response (Figure 4b) is very weak and not a good indicator of sample permeability. For this reason, the phase response is not considered here and has been omitted from the results in the remainder of this paper. It is worth noting that other EM systems such as EMspec [22] use a differential pair of receiver coils, where one receiver is positioned in the vicinity of the sample strip and another receiver further away. Such differential systems can provide a valuable phase response; however, they can be more sensitive to mechanical vibration. This study uses a simple transmitter—receiver configuration to demonstrate potential, but there is clearly scope for future refinement exploiting both phase and amplitude information, but that was not the purpose of this study.

It can be seen from the amplitude plot (Figure 4a) that the response at lower frequencies is very weak, and closer examination of the plot indicates that any frequency below 500 Hz would be unlikely to yield coherent results; therefore, 500 Hz was selected as the lowest excitation frequency. The amplitude response shows a peak at approximately 40 kHz, due to the roll-off for the frequency response of the amplifier. This frequency also gives the greatest difference between the three samples; consequently, 39.7 kHz was chosen as the highest excitation frequency. Two more intermediate frequencies were selected, resulting in the following frequency spectrum, with applied current weighting shown in brackets: 0.5 kHz (60%), 4.7 kHz (20%), 21.1 kHz (10%) and 39.7 kHz (10%). The current weighting is used to partially compensate for the weaker signal at lower frequencies; it is not intended to bring all the signals to exactly the same level.

**Figure 5:**
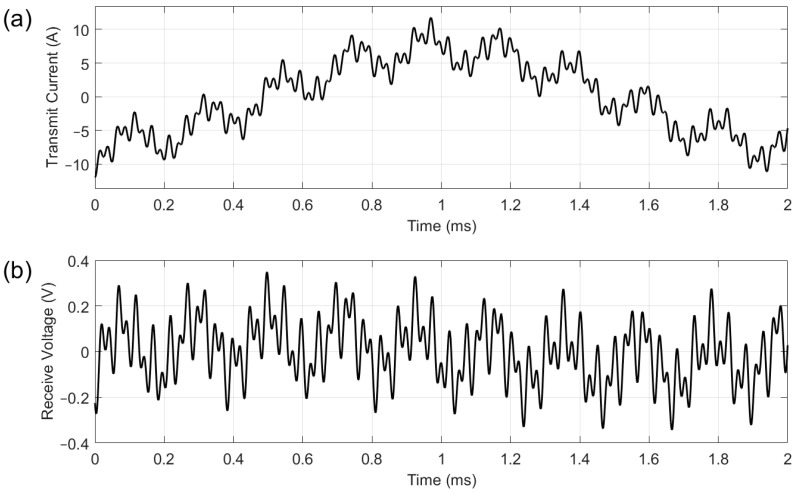
Current applied to the transmit coil (**a**) and voltage recorded by the receive coil (**b**).

Figure 5 shows the resultant multifrequency transmit current and receive voltage for the system. The *x*-axis shown here corresponds to one cycle of the 500 Hz waveform, though 50 cycles are acquired for FFT analysis during testing. It can be seen from Figure 5a that the excitation uses the full ± 12 A current range of the power amplifier, with a clear contribution from the 500 Hz component. The amplitude of signal from the receive coil shown in Figure 5b is approximately ±350 mV, although a gain of 10 is applied to this before data acquisition. For the received voltage, the higher frequencies are dominant.

### 2.3. Materials and Experimental Procedure

**Figure 6:**
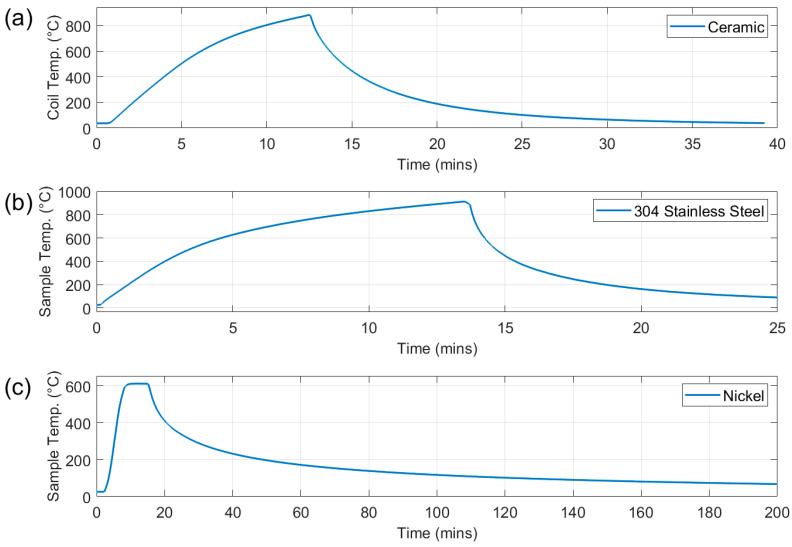
Heating and cooling cycles for ceramic plate (**a**), type 304 stainless steel (**b**), and nickel (**c**).

Three different measurements were carried out on the test rig using the same sample size with some variation in thickness. Although the sample holder can accommodate a sample measuring 225 mm × 80 mm, a smaller size of 100 mm × 80 mm was chosen for these tests to ensure uniform heating. Firstly, a 3 mm thick ceramic plate was used as a dummy sample to assess the level of background signal in the absence of a metal sample and provide data for background signal subtraction. For this test the sample was loaded into the furnace; the furnace was heated to 900 °C for austenitic steel and ferritic stainless calibration whilst 600 °C was used for nickel calibration test. Once the coil reached the target temperature, the sample holder was lowered out of the furnace and air cooled. The heating and cooling cycle for the test to 900 °C test is shown in Figure 6a.

The second test used a 1 mm thick type 304 austenitic stainless-steel plate. As this material is paramagnetic, it provides an assessment of the response of the system to a change in resistivity without an accompanying change in magnetic permeability. For the 304 stainless steel the furnace was heated to 400 °C; the sample was loaded into the furnace; the furnace was then heated to 900 °C with an average sample heating rate of 0.76 °C/s; once the sample temperature reached 900 °C/s the sample holder was lowered out of the furnace and allowed to air cool to <100 °C. See Figure 6b.

A 1 mm thick 99% pure nickel plate was employed in the third test to assess the response of the system to changes in resistivity and magnetic permeability. Nickel was chosen as it has a relatively low Curie temperature (Tc) at 353–360 °C, so it presents the opportunity to also observe the accuracy of the system to characterise the ferromagnetic to paramagnetic transition at Tc. For this test the furnace was heated to 200 °C; the sample was loaded into the furnace; the furnace temperature was increased to 600 °C with an average heating rate of 1.67 °C/s; once the sample temperature reached 600 °C, the furnace was switched off, allowing the sample to cool to <100 °C. See Figure 6c. The slow furnace cooling rate was chosen to assess the system’s ability to accurately determine the Tc of nickel during cooling.

The fourth test used a 0.9 mm thick type 430 ferritic stainless steel plate to assess the performance of the system when presented with a sample with a higher permeability and a higher temperature Curie point. This is more representative of the high-temperature behaviour that might be encountered in continuous annealing. The heating and cooling routine was identical to the test for the austenitic stainless steel shown in Figure 6b.

Type K thermocouples were spot-welded to the sample surface in the centre of the coil (middle of the sample) and directly opposite. A type K thermocouple was also embedded into the coil assembly to measure the temperature of the coil. It should be noted that this thermocouple did not contact the copper coil directly; it was sandwiched between the turns of the coil outside of the insulation. For the ceramic dummy sample, EM data is plotted against the coil temperature. For the stainless steel and nickel, the temperature of the surface of the material at the centre of the coil is used.

## 3. Finite Element Modelling

**Figure 7:**
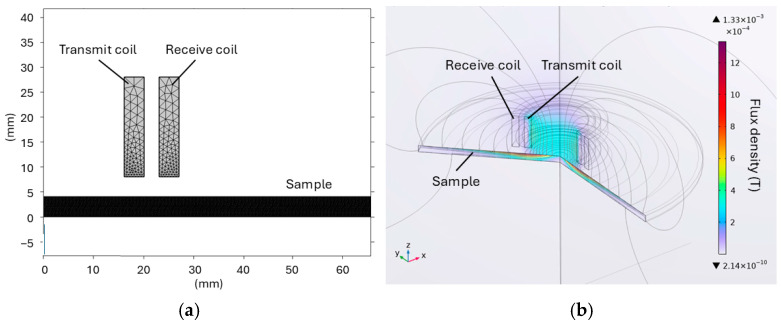
Two-dimensional, axisymmetric FEM model with geometry of the sensor and sample (**a**) and magnetic flux density distribution by rotating the modelled plane 360 degrees (**b**). μ = 230, frequency = 500 Hz, current = 2 A.

In order to gain insight into the interaction between the field and sample at the chosen frequencies, a finite element model was constructed. A two-dimensional, axisymmetric model was developed using COMSOL Multiphysics (Version 6.3). The exterior boundaries were set as magnetic insulation, and the interior boundaries were set as continuity. The complete mesh consists of 61,501 domain elements and 1240 boundary elements. The geometry of the sensor and sample in the model is shown in Figure 7a. Figure 7b shows the magnetic flux density distribution by rotating the modelled plane by 360 degrees.

**Figure 8:**
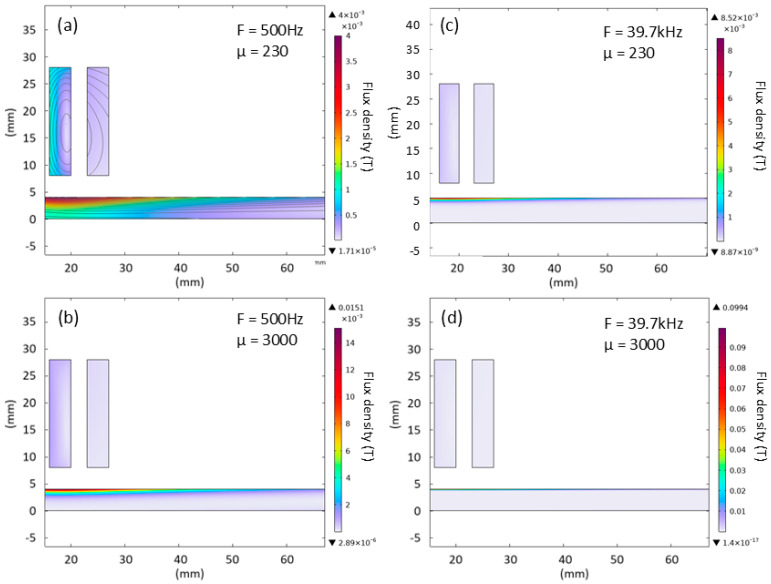
Flux density in the sample at low (**a**,**b**) and high (**c**,**d**) frequency excitation for two different permeability values.

Figure 8 illustrates how permeability and frequency affect the depth of magnetic field penetration into the steel plate. As either permeability or frequency increases, the skin depth becomes shallower, the induced field becomes more concentrated under the coil, and the maximum flux density at the sample surface increases. Hence, the higher frequencies offer a more localised measurement and a stronger response at the expense of sample penetration.

**Figure 9:**
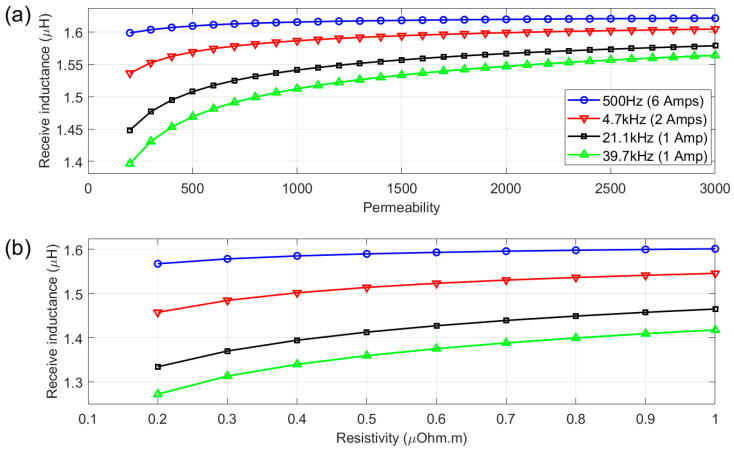
Magnitude of receive coil inductance for the chosen excitation frequencies and corresponding currents used in the experimental work for a variation in relative magnetic permeability (**a**) and resistivity (**b**). Fixed permeability of 500 used for (**b**).

As shown in Figure 9a, the modelling shows a monotonic increase in the receive coil inductance with increasing permeability across all frequencies. As frequency increases, so does the sensitivity to permeability changes, indicating that the highest frequency should result in the greatest sensitivity to a temperature-induced change in permeability in a ferromagnetic material. Conversely, the 500 Hz plot is relatively flat across all permeability values, indicating a poor correlation with permeability at this frequency. Figure 9b shows the receive coil inductance for a fixed permeability and a change in resistivity. It can be seen from the plot that the higher frequencies have the highest sensitivity to the change in resistivity, with 21.1 kHz and 39.7 kHz showing a very similar overall change.

## 4. Experimental Results

### 4.1. Ceramic Dummy Sample (Air Measurement)

**Figure 10:**
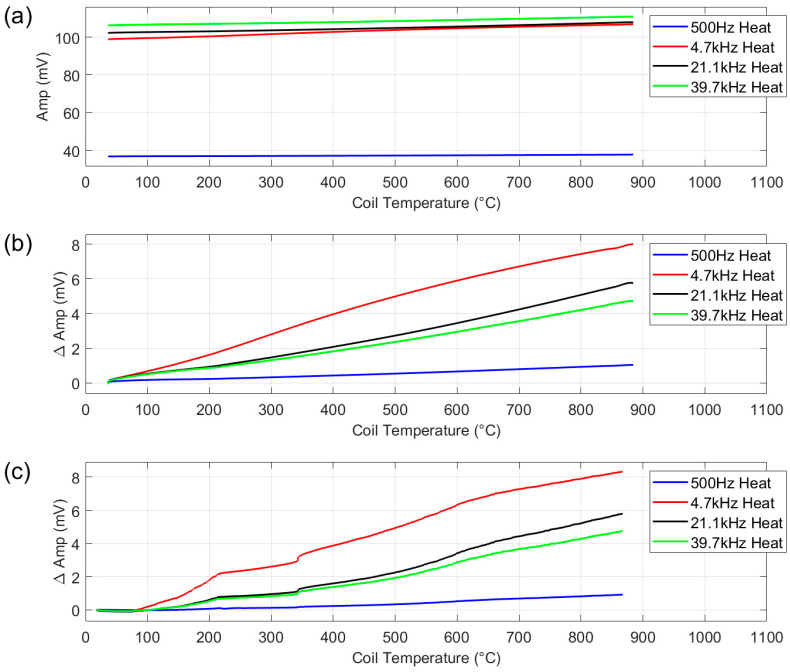
Multifrequency amplitude measurement for ceramic dummy sample in sample holder—absolute amplitude (**a**), multifrequency amplitude measurement for ceramic dummy sample in sample holder—change in amplitude (**b**) and multifrequency amplitude measurement for coil in furnace without sample holder (**c**). Represents the background signal of the system at the four chosen frequencies.

Figure 10a,b show the results of the test with the ceramic dummy sample. Figure 10a shows the absolute amplitude values, whereas Figure 10b shows the change in amplitude from the start of the test to allow comparison of the different frequencies on the same plot. The remainder of the figures in this paper are shown in the same format.

As the ceramic sample is not electromagnetically reactive, the measurement with the ceramic sample can be considered an air measurement. The ideal response for an air measurement in this scenario would be zero change in signal for the increase in temperature, indicating zero background field. However, a small change in amplitude is evident for all frequencies. It can be seen from the plot that the increase in amplitude on heating is almost linear with respect to coil temperature (*x*-axis), with 500 Hz showing the lowest overall change in amplitude, 4.7 kHz the highest and the other two higher frequencies lie between the two.

To assess if the interaction between the applied field and the austenitic stainless steel sample holder could be the source of the background signal, the test was repeated with the coil winched into the furnace without the sample holder, suspended from ceramic fibre cable, see Figure 10c. As the coil was not held in position by the sample holder in this test and could move freely in the furnace, the results are noisier; however, the trends in the data in Figure 10b,c are the same, indicating that the sample holder is not the source of the background signal.

The system is current-driven; therefore, the applied current does not vary with changes in the resistivity of the coil, so this can also be ruled out as the source of the background signal. As these factors have been eliminated, the background signal measured in this test must be caused by the generation of eddy currents in the 4 mm thick copper coil. It is expected that this background signal could be removed with future improvements in the system such as a balance differential coil configuration.

### 4.2. Austenitic Stainless Steel

**Figure 11:**
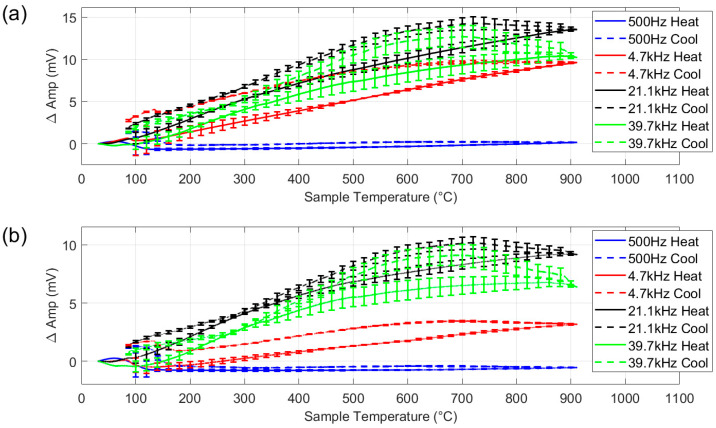
Multifrequency amplitude measurement for austenitic stainless steel (**a**), with background signal subtracted (**b**). With 1SD error bars.

Figure 11a shows the amplitude response to a sample made from type 304 austenitic stainless steel. This is nominally a fully paramagnetic material; therefore, magnetic permeability should remain constant, while resistivity increases with temperature, increasing the measured signal amplitude.

Examination of Figure 11a shows that at the lowest frequency (500 Hz), the change in amplitude is minimal, indicating limited sensitivity to the thermally induced change in resistivity. As frequency increases, the sensor becomes increasingly responsive: the higher frequencies show a notable rise in amplitude and with temperature. On cooling, the signal amplitude increases slightly before decreasing. Examination of the material by electron backscatter diffraction showed trace amounts of retained martensite, which indicates that the material was weakly ferromagnetic before heating and after cooling, so the response here cannot be seen as purely paramagnetic. However, these effects are small in magnitude and secondary to the main focus of this work.

Figure 11b shows the response from the stainless steel with the air measurement shown in Figure 10b subtracted. Both data sets include a coil temperature measurement. The coil temperature measurements for the austenitic steel were compared to the coil temperature measurements for air, and the corresponding air amplitude values were subtracted. It can be seen from the plot that the air subtraction causes an overall reduction in amplitude, around 50% decrease for the two higher frequencies. As the austenitic steel sample has a relatively low room temperature conductivity (1.39 MS/m in comparison with 58.5 MS/m for the copper coil [27]), the signal from the sample is relatively weak, so the background signal from the copper coil tends to dominate, distorting the austenitic steel response, especially on cooling.

### 4.3. Pure Nickel

**Figure 12:**
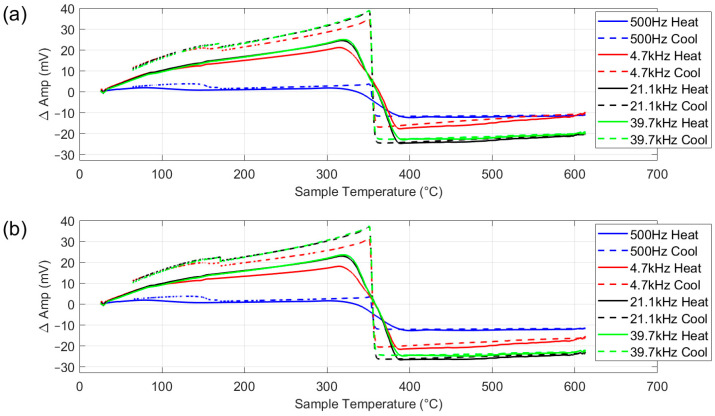
Multifrequency amplitude measurement for pure nickel (**a**), with background signal subtracted (**b**).

Figure 12a,b show the electromagnetic response to the heating and cooling of the nickel sample with and without background signal subtraction. Comparison of the two plots shows that background signal subtraction has very little impact, with some small shifts in the overall signal level at higher temperatures. As nickel has a Tc of 353–360 °C, a strong response to the ferromagnetic to paramagnetic transition is expected in this range. As with the austenitic steel (Figure 11), the higher frequencies give the sharpest response, this is attributed to increased sensitivity to near-surface properties due to a shallower penetration depth, as illustrated by the simulation results, though the 500 Hz plot does clearly reflect the Curie transition on heating and cooling.

The initial increase in amplitude at lower temperatures can mainly be attributed to the increase in magnetic permeability with temperature, with some contribution from the increase in resistivity, as with the austenitic steel discussed in Section 4.2. As temperature rises, the permeability increases, enhancing the interaction with the electromagnetic field, which results in a higher induced voltage and, consequently, a higher amplitude signal. This trend continues until the material approaches Tc, where magnetic permeability drops sharply as the nickel transitions to a paramagnetic state. After that, further temperature increases invoke a small increase in amplitude as a result of a further increase in resistivity. On cooling, the process is reversed.

Closer examination of the strongest response, the 39.7 kHz plot, shows that on heating, the change in amplitude corresponding to the Curie transition is somewhat blurred, starting at around 325 °C and finishing at around 392 °C. This is caused by the relatively fast heating of the sample, 2.18 °C/s between 300 °C and 400 °C, meaning that different parts of the sample are heating at slightly different rates and reaching Tc at different times. In contrast, on cooling, the change in amplitude corresponding to the Curie transition takes place between 360 °C and 351 °C, much closer to the expected transition temperature, reflecting the much slower cooling rate of 0.2 °C/s.

### 4.4. Ferritic Stainless Steel

**Figure 13:**
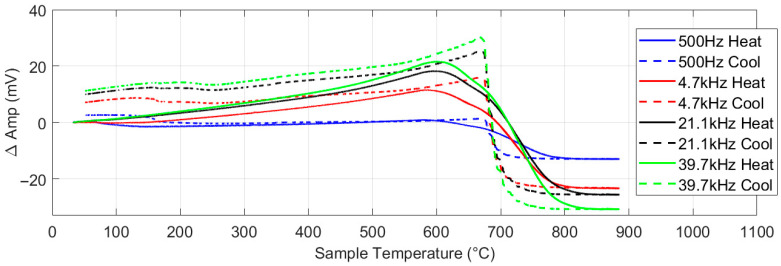
Multifrequency amplitude measurement for type 430 ferritic stainless steel with background signal subtracted.

Figure 13 shows the EM response for the 430 ferritic stainless steel sheet. As with the nickel, the higher frequencies give the strongest response. Examination of the heating part of the cycle for the 39.7 kHz plot shows that there is an initial rise in amplitude as permeability increases, followed by a drop in signal level as the temperature increases past 600 °C. This drop in signal level is due to parts of the sample reaching the Curie temperature, as the relatively high heating rate caused some temperature non-uniformity in the sample. After the temperature passes Tc for all the samples, the signal levels off, apart from a very small increase due to the continuing increase in resistivity. The Tc is more clearly seen on cooling as the low cooling rate results in less temperature gradients across the sample. The lower Tc value (approx. 700 °C) than seen in iron or low carbon steels (approx. 780 °C) is due to the high Cr content in the steel.

## 5. Discussion

This paper outlines work to develop an electromagnetic sensor system for deployment in a continuous annealing furnace. The sensor system must be capable of in situ characterisation of the material in the furnace, reliably tracking evolving microstructures and providing real-time feedback to ensure mechanical property targets are met. The sensor must be able to survive prolonged periods in an extreme environment at temperatures up to approximately 850 °C, depending on sensor positioning in the furnace. The system consists of three parts: the sensor assembly, custom-built hardware and software. In order to assess the system as a whole, a number of experiments were set up using an annealing simulator test rig which employs a vertical tube furnace for sample heating and cooling in conjunction with a winch to move the samples in and out of the furnace.

As can be seen in Figure 11, there is some variation, as indicated by the error bars, in the measured signal for repeated tests with the same sample. Some potential sources of error, including electrical noise from the furnace, measurement drift, variations in background signal and thermally induced changes in sensor geometry have been studied during the design stage and found to have negligible impact on the measured signal. The greatest potential source of error in this system is a variation in lift-off: the distance between the sensor and sample. Although, as shown in Figure 1 and Figure 2, the position of the coil is fixed with respect to the frame, the sample is permitted some movement to allow for thermal expansion. Thus, some lift-off variation is possible. Tests with the ferrite powder samples showed that the signal level for all frequencies decreased by a factor of ≈ 6 for an increase in lift-off from 2 mm to 5 mm. Although it is unlikely that lift-off could increase this much during normal testing, it does illustrate the potential for lift-off-related error. Previous work has shown that through judicious sensor design, careful frequency selection, analysis of the signal phase and calibration procedures, a lift-off invariant multifrequency EM measurement system can be realised [21,22]. This will be a goal for the next iteration of this system.

The tests did not use samples representative of the types of materials processed in a continuous annealing furnace, rather the samples were selected to interrogate specific heat induced phenomena: a ceramic dummy sample to assess the background signal in the first test (Figure 10); an austenitic steel sheet to assess the response of the signal to a change in electrical resistivity only in the second test (Figure 11); a nickel sample to assess the capability of the system to accurately characterise the ferromagnetic to paramagnetic Curie transition in the third test (Figure 12); a ferritic stainless steel sample to assess the performance on the system when presented with a higher temperature ferromagnetic to paramagnetic Curie transition in a high permeability sample in the fourth test (Figure 13).

The test results show that although the system does exhibit a significant change in background signal, this is repeatable and can be successfully subtracted from the sample signal. The result for the nickel sample shows a very accurate Curie transition on cooling, however the signal corresponding to the Curie transition for heating is spread over a much wider temperature range, due to variations in temperature across the sample at the relatively high heating rate (compared to cooling rate) and the large diameter of the coil measuring a large sample area. The result for the ferritic stainless steel shows that the system is capable of characterising changes at high temperatures, as would be found in a CAP line. As with the nickel sample, there are some discrepancies between the measured temperature and the expected Curie transition temperature, again due to the size of the coil in comparison with the sample, combined with the fast heating. The presence of the coil adjacent to the sample also induced some temperature non-uniformity in the annealing simulator system. The EM measurement system is ultimately intended for deployment in a much larger furnace and on much larger samples (industrial CAP line with wide strip samples that continuously move past the sensor), therefore in practice, the coil will not encounter these large thermal gradients.

The range of frequencies employed in this work was primarily dictated by the bandwidth of the system, with modelling and experimental work used to assess the suitability of each frequency for material microstructure assessment. Figure 4, Figure 8 and Figure 9 show experiment and modelling results that illustrate the sensitivity of the system to materials with different permeabilities at different frequencies. The data shows a monotonic increase in magnitude with increasing permeability across all frequencies. This behaviour reinforces the experimental strategy: as steel undergoes phase transformation during annealing (changing permeability), the signal amplitude responds consistently. At higher frequencies (21.1 kHz and 39.7 kHz), the magnitude shows more distinct gradients with respect to permeability; these frequencies induce stronger, more localised signals, with a greater sensitivity to surface-level changes due to shallower penetration depths. In contrast, 500 Hz provides deeper penetration but less sensitivity to permeability changes; however, the greater penetration depth may make this frequency more suitable for bulk behaviour interrogation.

The combined insight from the experimental work and modelling will inform frequency selection for specific transformation tracking in the continuous annealing furnace application. For example, detecting ferrite–austenite transitions (which alter magnetic permeability) may benefit from multifrequency interpretation, using both deep and shallow probing characteristics. The high frequency magnitude shows the highest sensitivity to permeability changes, but if there is a surface effect on the material, low frequency data can be used to analyse through-thickness variation. Surface effects in steel can include phenomena such as decarburisation, where a lower carbon content at the surface can cause earlier transformation to ferrite on cooling than the bulk material and a higher permeability in that surface layer. If surface effects are not an issue, it may be judicious to discard the 500 Hz data, as it accounts for 60% of the applied current, and concentrate more energy in higher frequency bands.

In order to reach a higher environmental temperature, the sensor design is much simplified in comparison with other high-temperature EM measurement systems [5,21,22], leaving the signal more vulnerable to noise sources. For example, [21] uses a ferrite core to shape the applied field and increase maximum lift-off and a dummy sensor to subtract the background field. Although the same techniques cannot be applied to this system as the core would not survive, a similar approach can be adopted by employing a differential receiver coil configuration with associated signal processing to measure only the signal from the target steel strip. This would allow more sophisticated processing of the steel properties than attempted here. Isolating the signal from the target also allows further calibration and removal of variation in lift-off. Along with sensor improvements, further investigations will involve real-time microstructure change tracking for advanced steels such as dual-phase and interstitial free grades, with sensor output calibrated to represent real material properties such as magnetic permeability.

## 6. Conclusions

This study establishes a proof of concept for an EM test system capable of real-time detection of magnetic and structural transformations in metals during thermal cycling in a high-temperature furnace environment. An annealing simulator test rig was used to test a range of samples selected to interrogate different aspects of the sensor response. The test results show a correlation with the thermally induced change in conductivity in an austenitic stainless steel sample and a strong response to increasing permeability below Tc and the subsequent ferromagnetic—paramagnetic Curie transition in nickel and ferritic stainless steel. The sensor employs a simplified design in comparison to other sensors of this type, using a few turns of 4 mm thick copper rod for the transmit coil and high-temperature type K thermocouple wire for the receive coil, resulting in a robust temperature-resistant construction. The developed sensor assembly has proved to be tolerant to high-temperature deployment, surviving more than twenty tests where the sensor was exposed to thermal cycling up to 900 °C and one test where the sensor was heated to 715 °C and held at that temperature for five hours without significant damage. Future iterations of the sensor design will feature a differential receive coil configuration to aid in background signal subtraction and further investigation of the multifrequency phase response to mitigate the effects of lift-off variation on the measured signal. The eventual aim of the work is sensor deployment in an industrial continuous annealing line, providing real-time feedback for precise processing control in the production of high-value steels.

## Data Availability

Data supporting the conclusions of this article will be made available by the authors upon request.

## Abbreviations

The following abbreviations are used in this manuscript:

CAP	Continuous Annealing Process
EM	Electromagnetic
IMPOC	Impulse Magnetic Process Online Controller
HACOM	Harmonic Analysis Coil Online Measuring
3MA	Micromagnetic Multi-parameter, Microstructure and Stress Analysis
Tc	Curie Temperature
BOP	Bipolar Operational Power supply
